# Impact of Ivermectin on the Gut Microbial Ecosystem

**DOI:** 10.3390/ijms242216125

**Published:** 2023-11-09

**Authors:** LinShu Liu, Karley K. Mahalak, Jamshed T. Bobokalonov, Adrienne B. Narrowe, Jenni Firrman, Johanna M. S. Lemons, Kyle Bittinger, Weiming Hu, Steven M. Jones, Ahmed M. Moustafa

**Affiliations:** 1 Dairy and Functional Foods Research Unit, Eastern Regional Research Center, Agricultural Research Service, United States Department of Agriculture, Wyndmoor, PA 19038, USA; 2 Division of Gastroenterology, Hepatology, and Nutrition, The Children’s Hospital of Philadelphia, Philadelphia, PA 19104, USA; 3 Department of Pediatrics, Perelman School of Medicine, University of Pennsylvania, Philadelphia, PA 19104, USA

**Keywords:** ivermectin, gut microbiome, short chain fatty acid, SHIME, soluble fiber, insoluble fiber

## Abstract

Ivermectin is a an anti-helminthic that is critical globally for both human and veterinary care. To the best of our knowledge, information available regarding the influence of ivermectin (IVM) on the gut microbiota has only been collected from diseased donors, who were treated with IVM alone or in combination with other medicines. Results thus obtained were influenced by multiple elements beyond IVM, such as disease, and other medical treatments. The research presented here investigated the impact of IVM on the gut microbial structure established in a Triple-SHIME^®^ (simulator of the human intestinal microbial ecosystem), using fecal material from three healthy adults. The microbial communities were grown using three different culture media: standard SHIME media and SHIME media with either soluble or insoluble fiber added (control, SF, ISF). IVM introduced minor and temporary changes to the gut microbial community in terms of composition and metabolite production, as revealed by 16S rRNA amplicon sequencing analysis, flow cytometry, and GC-MS. Thus, it was concluded that IVM is not expected to induce dysbiosis or yield adverse effects if administered to healthy adults. In addition, the donor’s starting community influences the relationship between IVM and the gut microbiome, and the soluble fiber component in feed could protect the gut microbiota from IVM; an increase in short-chain fatty acid production was predicted by PICRUSt2 and detected with IVM treatment.

## 1. Introduction

Ivermectin (IVM) is a macrocyclic lactone produced via fermentation by the soil microorganism *Streptomyces avermitilis* [1,2]. IVM has demonstrated potent activity in combating a broad spectrum of intestinal helminthic parasites and ectoparasites [3,4] and is highly effective in treating filarial diseases and controlling malarial diseases [5,6,7,8]. As it can be used to treat both humans and animals, its application has improved the quality of life for billions of people living in underdeveloped areas and increased the profitability of the global livestock industry [9,10,11].

Several mechanisms of action have been reported for IVM, which include activating the glutamate-gated chloride channel receptors and causing cell hyperpolarization, disrupting central nervous system neurosynaptic transmission, and inhibiting the secretion of extracellular vesicles from parasites [12,13,14]. Recent research revealed that IVM may inhibit the lipopolysaccharide-induced production of inflammatory cytokines via the inhibition of importin α/β, a pathway of protein transport into the nucleus. It is also reported that ivermectin targets host inflammatory pathways like nuclear factor kappa-light-chain-enhancer of activated B cells (NF-κB), Akt kinase/mammalian target of rapamycin (AKT/mTOR), and signal transducer and activator of transcription 3 (STAT3) [15,16,17,18,19]. These mechanistic studies facilitated research on re-purposing applications of IVM as a cancer therapeutic [20,21,22] and as a treatment for infectious diseases caused by bacteria, fungi, and viruses [23,24,25,26,27]. Broadening the application potential of IVM to treat diseases and infections beyond its current use increases the importance of understanding the effect of IVM on human biology—in particular, its ability to modulate the commensal microorganisms that reside within the gastrointestinal tract (GIT). Furthermore, oral delivery is the most effective and convenient method of IVM administration for the treatment of colonic or systematic diseases, and, with this delivery method, IVM has direct interactions with the gut microbiome in addition to the treatment target.

Ideally, in addition to being an effective therapeutic, short-term IVM administration should not largely alter the composition of the host microbial community, as these changes could lead to dysbiosis, which puts the individual at risk of further disease development. However, IVM has been reported to change the gut microbiota of diseased patients. For example, the abundance of the phylum Bacteroidetes increased in stool samples of hookworm-infected adolescents 24 h after the administration of IVM [28,29], but this was reversed three weeks post-treatment. The results of this study also found that the composition of the starting bacterial community impacted the success of anti-helminthic treatment. A small-scale experiment on the deer *Cervus nippon* found that IVM had more significant effects on the fungal communities than on bacterial communities [25]. However, all these investigations were carried out on subjects undergoing anti-helminthic treatment, which confounds the effect of IVM and makes it impossible to decipher between changes elicited due to IVM treatment and those resulting from the helminth infection. Furthermore, these tests were all performed in vivo, which makes it impossible to elucidate the effect of IVM on the gut microbiota specifically, as it is well known that the mammalian and microbial cells interact metabolically [30]. To date, an investigation of IVM’s effect on the gut microbial community of healthy individuals has not been conducted [31], even though such studies could provide important safety information for those considering re-purposing IVM for alternative applications in which the subjects are not also infected with helminths.

In the present research, the effect of IVM on the gut microbiota structure and function was analyzed using DNA marker gene sequencing coupled with metabolomics. For this study, an in vitro platform was specifically applied to provide direct answers as to how IVM may affect the gut microbiota while eliminating interference from host cells or the interplay between microbial and host cells that would occur in vivo [32,33]. Since reports have indicated IVM as an antimicrobial agent capable of eliciting changes to the gut microbiomes of diseased patients, the ability for nutritional supplementation to mitigate microbiome alterations due to IVM was also tested using fibers known to promote the health of the gut microbiota. Functional changes to the microbiome in response to IVM were assessed, and whether the metabolites produced would impair mammalian cell function along the GIT was tested using the in vitro cultivation of CACO2 cells. Together, these results provide valuable information on the effects of IVM on the gut microbiome in terms of structure, metabolic output, and its impact on cellular function in the GIT.

## 2. Results and Discussion

### 2.1. IVM Administration Increased Cell Mortality in the Proximal Colon Region

**Figure 1:**
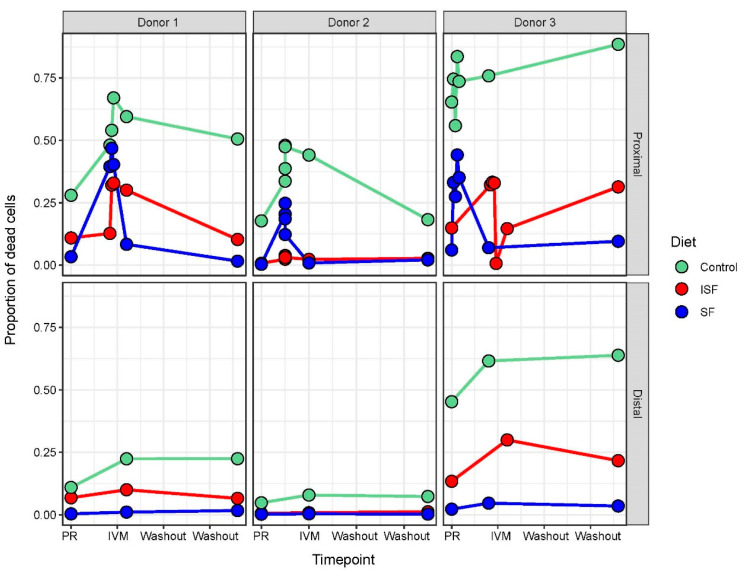
Flow cytometry indicating proportion of dead cells for communities with the proximal and distal colon regions during pretreatment, IVM treatment, and washout periods.

Although IVM is used to treat helminth infections, there have been reports that it can also function as an antimicrobial agent [34,35]. To first determine if IVM exhibited antimicrobial activity in the context of the gut microbiota, flow cytometry was performed to measure the proportion of dead to live bacterial cells, before, during, and after IVM addition (Figure 1). Immediately after addition, there was a large jump in cell mortality in the proximal colon communities, but less in the distal colon communities, for all three nutritional formulations tested (control, ISF, and SF). This observation was logical as IVM was added directly only to the proximal colon, and the distal communities only received IVM indirectly, as the fluid within the proximal colon was transferred to the distal colon region during feeding. Cell numbers stabilized after this initial addition and, in some cases, even started to recover. Notably, the proportion of dead bacterial cells measured in the cultures provided SF nutrition was much less than in ISF, and losses in both SF and ISF were less than in the control communities.

The co-occurrence of IVM administration and increased cell mortality observed here followed previous findings that IVM exerts antimicrobial actions against some bacteria, such as *Mycobacterium*, *Chlamydia*, and *Staphylococcus* [34]. While these are not taxa canonically associated with the gut microbiota, the observation here indicated that the antimicrobial effects of IVM may extend beyond these taxa. Additionally, these results indicated that providing fiber in the nutritional formulation (SF and ISF) decreased mortality during IVM administration, which is consistent with recent research showing that fiber supplementation is protective against the effects of antibiotics [36]. Although the mechanism behind SF and ISF protection against IVM-mediated death was unclear, this suggested that the effects of IVM on the gut microbial community may be mitigated using fiber prebiotics in conjunction with IVM administration.

### 2.2. Effect of IVM on the Gut Microbiota Structure in Terms of Alpha and Beta Diversity

**Figure 2:**
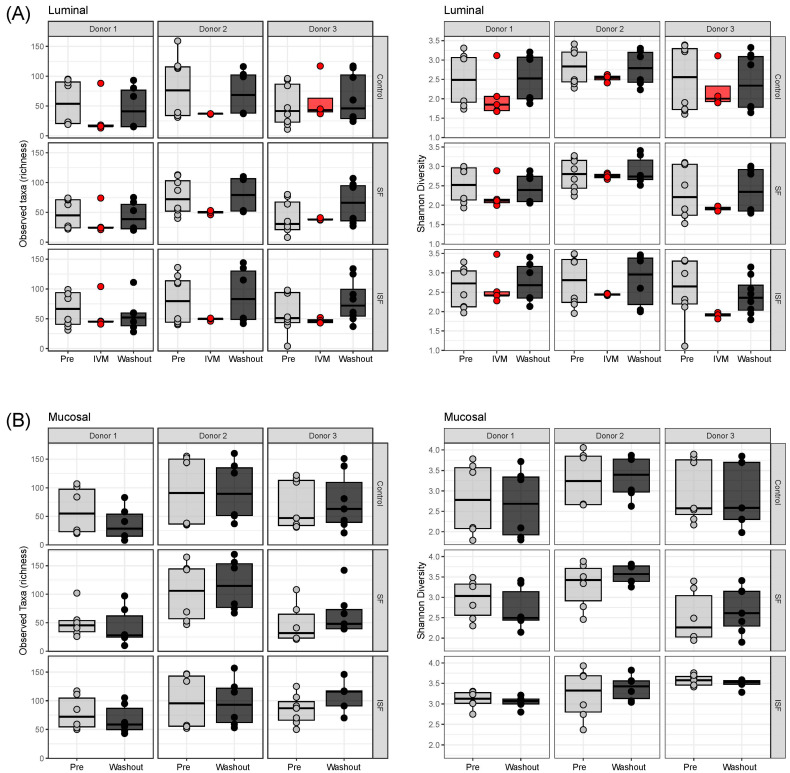
Alpha diversity in terms of richness, determined by number of observed taxa and evenness, determined by the Shannon Diversity Index, for the (**A**) luminal and (**B**) mucosal communities. Data from the proximal and distal colon communities were combined for the control, SF, and ISF nutritional formulations tested during the pre-treatment, IVM treatment, and washout phases. Open circles on boxplots indicate individual samples.

To further evaluate this observed effect, 16S rRNA sequencing was used to examine the microbial community structure comparing both luminal and mucosal communities with and without IVM administration. To begin, the alpha diversity metrics of taxon richness and Shannon’s Diversity Index were calculated for the luminal and mucosal communities (Figure 2). For the luminal phase, an obvious, but not significant, decrease in taxa richness and Shannon Index was identified upon the addition of IVM for the three donors; however, the numbers were recovered through the washout period (Figure 2A). As IVM was not injected in the mucosal phase, only pre-treatment and the washout period were analyzed, which revealed no significant differences in either taxa richness or Shannon’s Diversity Index (Figure 2B). The only statistically significant differences were seen by comparing the two different colonic regions (proximal vs. distal), where both the Shannon’s Index and taxa richness of the distal colon (DC) were higher than in the proximal (PC) region (Figure S1). Such differences have been observed before and can likely be attributed to the longer transit time in the DC than in the PC and other environmental differences between the two regions [37,38].

Next, changes to community structure driven by IVM administration were analyzed using the weighted UniFrac distance metric and visualized by Principal Coordinate Analysis (PCoA).When communities provided with the control, SF, and ISF nutritional formulations for each donor were combined, it was clear that, for the luminal communities, donor 1 separated from donors 2 and 3, and that the proximal and distal colon communities were divergent from each other (Figure S2). This was also observed for the mucosal communities, although the separation was less apparent. This demonstrated that the communities between donors were distinct from each other (*p* < 0.0001, PERMANOVA, pairwiseAdonis package), and that the proximal and distal regions of the in vitro apparatus fostered the development of unique communities in terms of structure (*p* < 0.0001, PERMANOVA, pairwiseAdonis package). Considering the strong effects of the phase and region, within these respective groups, samples from all donors differed significantly from each other, indicating the importance of donor effects as well. Even when the different diet types were taken into consideration, the samples in the distal colon region clustered apart from the proximal colon region.

**Figure 3:**
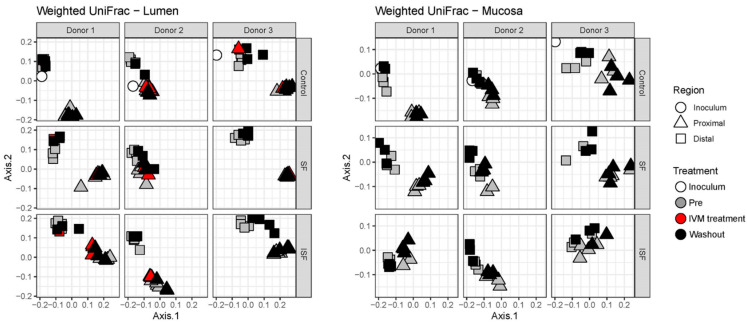
Weighted UniFrac distances portrayed as Principal Coordinate Analysis (PCoA) for the luminal and mucosal communities provided with control, SF, or ISF nutritional formulations during the pre-treatment, IVM treatment, and washout periods.

**Figure 4:**
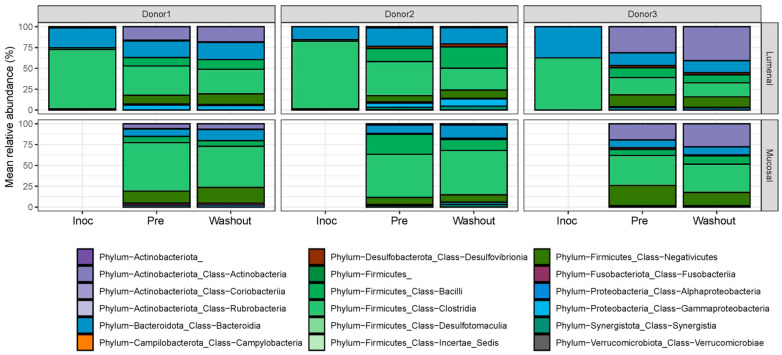
Bar plot depicting relative abundance of taxa within the communities at the class level based on 16S rRNA sequencing. Taxa are ordered by phylum membership.

Based on the results of the weighted UniFrac distances, there were no statistically significant changes detected based on the nutritional formulation or administration of IVM for either the luminal or mucosal communities (Figure 3). This indicated that any effects on the composition of the gut microbial communities were small in relation to the differences among donors and colon regions. This supposition was further supported through the examination of the specific compositions of the gut microbial communities at the phylum and class levels, which indicated no significant change in bacterial composition between the pre-treatment and washout periods for all three donors (Figure 4).

### 2.3. Effect of IVM on the Functionality of the Gut Microbiota

**Figure 5:**
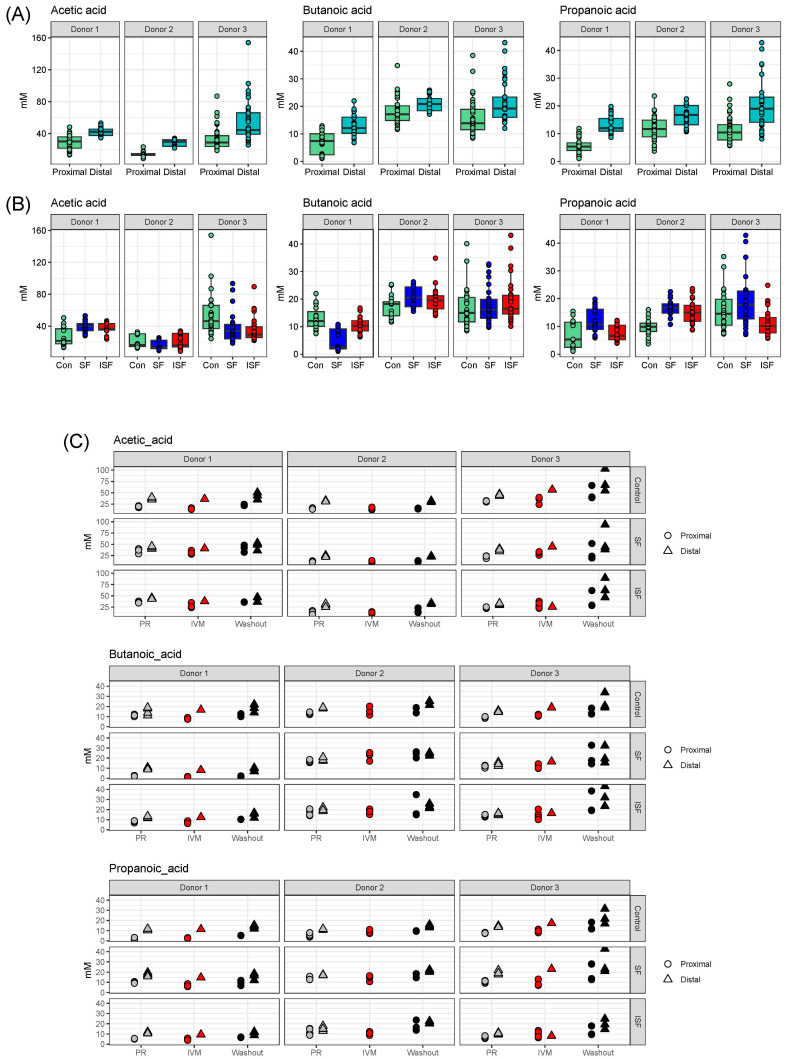
SCFA quantification looking at acetic, butanoic, and propanoic acid. (**A**) Levels of SCFAs in the proximal versus distal colon; (**B**) levels of SCFAs for all communities within the three different nutritional formulations (control, SF, and ISF) for each donor; (**C**) SCFAs detected in the proximal and distal colon regions for pre-treatment, IVM treatment, and washout phases cultured in each nutritional formulation.

The gut microbiota within the colon ferments undigested substrates that enter and releases corresponding metabolic byproducts, the most common of which are short-chain fatty acids (SCFAs). This functional role of the gut microbiota is of critical importance to the mammalian cells within the GIT, as well as cells throughout the human body. To determine whether IVM would alter this functional role, the three most prominent SCFAs were quantified, acetic, butanoic, and propanoic acids (Figure 5). When communities from all treatment phases were considered together, i.e., the pre-treatment, IVM treatment, and washout phases, the results showed that the measured amounts of these three SCFAs in the DC region were significantly higher than in the PC region for all three donors due to SCFA accumulation in the system (Figure 5A) [33]. When the effect of the provided nutritional formulations were also considered, there were some donor-dependent effects observed (Figure 5B). For donor 1, the fermentation of SF produced more acetate and propionate than from ISF and the control. In contrast, there were no significant differences in acetate and propionate production between ISF and SF for the other two donors. Butyrate production was significantly increased in the presence of SF/ISF only for donor 2.

When the SCFAs quantified within the different treatment phases were considered separately, the results showed that the timing relative to IVM treatment also impacted SCFA production (Figure 5C). In general, the amount of the three SCFAs measured in the washout period was statistically higher than in the pre-treatment control and IVM treatment groups. The highest increase in SCFAs in both the pre-treatment and washout period in both the PC and DC was detected for donor 3. This trend was not altered by the different nutritional formulations (Figure 5C).

**Figure 6:**
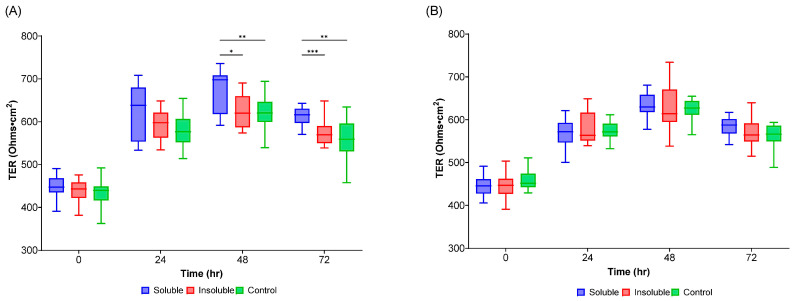
Transepithelial electrical resistance (TEER) of Caco2 cells incubated with supernatant harvested from the proximal region gut microbiota communities provided with control, SF, and ISF nutritional formulations (**A**) on IVM treatment day and (**B**) at washout. * *p*-value < 0.05; ** *p*-value < 0.01; *** *p*-value < 0.001.

Some metabolites produced by the gut microbiota, like SCFAs, are known to positively influence the barrier function of the host intestinal epithelium. It was considered that, although IVM may not have elicited large structural changes or altered the levels of acetic, butanoic, or propanoic acid, it may have stimulated the release of metabolites that would affect the barrier function of the host epithelium. To test this hypothesis, Caco2 cells were incubated with metabolites released from communities on the day of IVM treatment and in the middle of the washout, cultured in the control, SF, and ISF nutritional formulations. The barrier function of the cell layer was monitored by measuring transepithelial resistance (TEER) over 72 h (Figure 6). On the IVM treatment day, supernatants from the bacterial cultures on the SF nutritional formulation significantly increased the barrier function of the Caco2 cell monolayer over time (Figure 6A). This was not true of the supernatants from SF bacterial cultures during the washout period (Figure 6B). In other words, the TEER value induced by the SF supplementation was significantly less during the washout phase than on the day of IVM treatment for time points 24 h (adjusted *p*-value = 0.0305) and 72 h (adjusted *p*-value = 0.006); the 48-h time point did not quite reach significance (adjusted *p*-value = 0.0555). Supernatants from the control and ISF groups did not increase barrier function on either day and were not statistically different between the IVM treatment day and the washout timepoint.

These results suggested that the gut microbial communities grown in the presence of SF produced and excreted a compound that increased the barrier function of intestinal epithelial cells, but that treatment with IVM abrogated its production. We explored whether this compound comprised SCFAs, but while the total SCFAs produced in the soluble treatment on day 1 appeared higher than for the other two media, the difference was not significant (Figure S3). It is possible that the increased barrier function in cells treated with SF bacterial supernatants results from a factor other than SCFAs, perhaps an unknown microbial metabolite.

### 2.4. Predicting Functional Pathways

**Figure 7:**
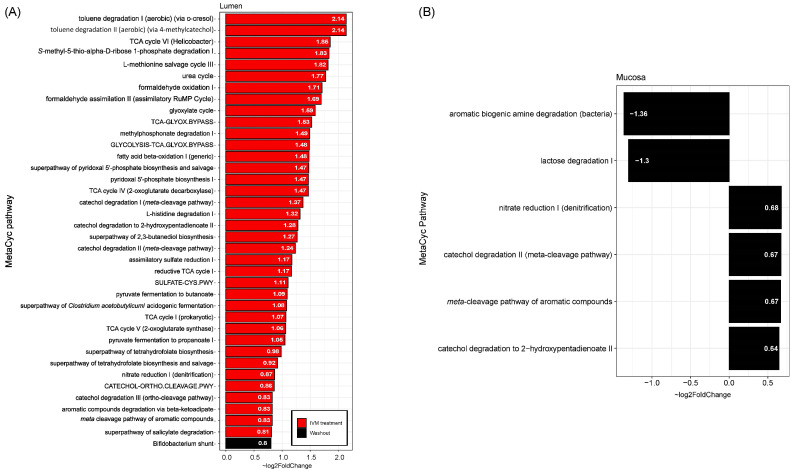
Log fold change in estimated MetaCyc function abundances that differed significantly in either IVM treatment or washout relative to pre-treatment. (**A**) Changed function in luminal samples; (**B**) changed function in mucosal samples.

Although IVM elicited only minor changes to the structure and function of the gut microbiota, it was considered that IVM may alter the functional potential of the communities. Therefore, the effect of IVM on microbial community functional potential was predicted using PICRUSt2 and the community composition was described as the estimated abundances of MetaCyc pathways, and genes classified by EC numbers. MaAsLin2 was used to identify pathways that differed significantly in estimated abundances in either the IVM treatment or washout samples relative to the pre-treatment communities. This analysis was conducted separately for the luminal and mucosal phases but did not separate between DC and PC. Donor and diet type were used as random effects. The results of this analysis identified 39 MetaCyc pathway abundances in the luminal phase and four in the mucosal phase that were significantly increased relative to pre-treatment, and two in the mucosal phase were significantly decreased (Figure 7). Pyruvate fermentation to propanoate (*q* = 0.0014) and pyruvate fermentation to butanoate (*q* = 0.024) are responsible for SCFA production [39,40,41], and these increases agree with the observed SCFA production (Figure 5).

**Figure 8:**
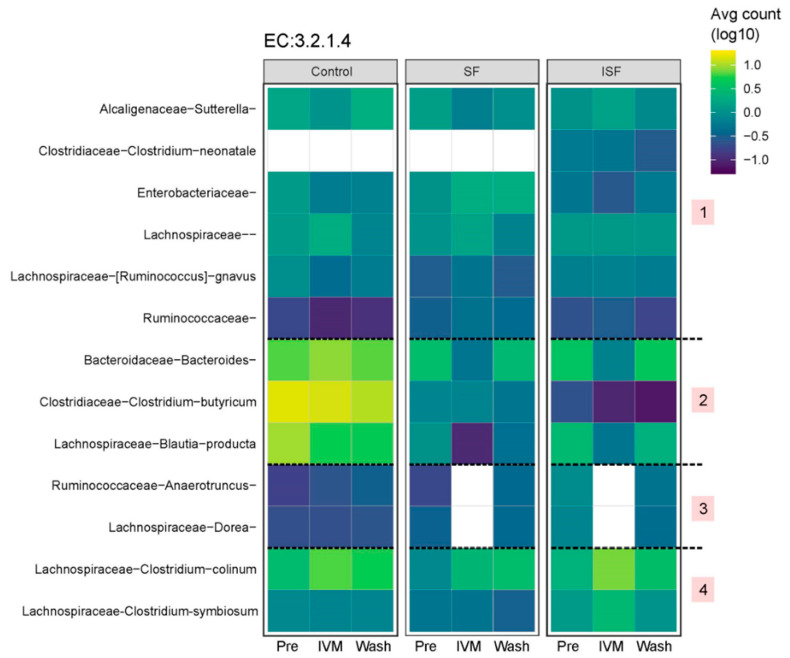
Estimated abundances of cellulase EC 3.2.1.4. Numbers in pink squares relate to Figure S4.

Finally, given the protective effects of fiber on the gut microbial community, and considering the important role that gut bacteria play in nutrient acquisition from fiber indigestible by the host, the effect of IVM on taxa containing key fiber-degrading functions was examined. Species predicted to contain cellulases active on 1,4-glycosidic bonds were identified from the PICRUSt EC results [42,43,44,45,46]. Figure 8 shows how the combined estimated abundances of one of these cellulases (EC:3.2.1.4) changed with the fiber types and the stages of IVM treatment. Cellulases of species from the genus *Alcaligenaceae sutterella* appeared to decrease in association with IVM treatment for the control and SF, but increased for ISF, and all three cases were recovered in the washout period. Similar examples were *Enterobacteriaceae* and *Clostridium colinum* for the control and ISF, *Blautia producta* for SF and ISF, and *Bacteroides* for all diet types. For other cases, some taxa’s cellulase abundances were reduced at the IVM treatment day and did not return to earlier levels, such as *Blautia producta* and *Clostridium butyricum* for the control and *Clostridium neonatale* for ISF. Figure S4 depicts the abundance of these bacteria by donor, colon region, and diet, showing specific effects of these factors. Species from *Lachnospiraceae* were higher in the proximal colon region of donor 3 with ISF and donor 2 with the control than the correlatives in the distal control region, but no difference was detected for donor 1 in ISF and donor 3 in the control. Species from *Clostridium butyricum* were higher in the proximal colon region of donor 1 with the control than in the distal colon region, but the opposite phenomenon was shown with SF and ISF. These results revealed the donor-dependent features of the gut microbial structure under the influence of not only IVM, but also the diet type [36,43,47], colon region, and stage of treatment.

## 3. Materials and Methods

### 3.1. Materials

Adult MSHIME medium with added starch was used as the Defined Medium (DM) for this experiment, purchased from Prodigest (Ghent, Belgium) and dissolved in MiliQ water at 19.6 g/L before autoclaving, as per the manufacturer’s guidelines. Soluble and insoluble fractions were procured from stabilized rice bran (RiceBran Technologies; The Woodlands, TX, USA) and 5 g/L of each was mixed with the DM (14.6 g/L) and termed SF (soluble fraction) and ISF (insoluble fraction), respectively. Pancreatic juice was produced from 12.5 g/L NaHCO_3_ (Sigma-Aldrich, Saint Louis, MO, USA), 6 g/L bile salts (BD, Franklin Lakes, NJ, USA), and 0.9 g/L pancreatin (Sigma-Aldrich, Saint Louis, MO, USA). Mucin agar was prepared using 5% type II porcine mucin (Sigma-Aldrich) and 1% bacterial agar in sterile MilliQ water, as described previously [33,48]. Fecal samples were obtained from OpenBiome (Boston, MA, USA). Donors were between 21 and 45 years of age, BMI < 30 [33,49], free of antibiotics for at least 1 year, consuming a standard Western diet, and matched all requirements that are used for blood donors [33].

### 3.2. Triple SHIME^®^ In Vitro Experiment

The Triple SHIME^®^ (Prodigest, Ghent, Belgium) was set up and operated as previously described [48,49,50], with minor modifications. Each experimental run of the SHIME used 1 individual donor fecal sample for inoculation. For each run, there were three “colon” sets (ergo, triple-SHIME) for the control (DM), SF, and ISF, separately. As depicted in Figure S5, all reactors were connected in sequence to the stomach and small intestine (ST/SI), proximal colon (PC), and distal colon (DC), for three total digestive systems. For each run, the experiment was performed for three weeks to establish and confirm a stabilized gut microbial community, and then 9 mg IVM was added to each proximal colon reactor (800 mL volume) to simulate taking IVM on an empty stomach and then run for an additional week. The IVM clinical dosage is determined by the patient’s body mass and the treatment target. The amount added was based on a recommended dosage of 12 mg/75 kg [51], with a 30% reduction based on experiments showing that 30% of the IVM is eliminated in the small intestine [52]. The one-week run time followed previously published timelines using the SHIME [50,53]. Samples were taken at regular intervals throughout the experiment, separated as bacterial pellets and bacteria-free supernatant, and stored at −80 °C for analysis [33].

### 3.3. 16S rRNA Sequencing

DNA was extracted and analyzed at the Microbiome Center, Children’s Hospital of Philadelphia (CHOP), as described previously [54]. The PowerSoil Pro DNA extraction kit (Qiagen, Hilden, Germany) was used for DNA extraction; the Illumina Miseq (San Diego, CA, USA) with a 2 × 250 bp reagent kit was applied for DNA sequencing. Libraries were generated, targeting the V4 region of the 16s rRNA gene [54].

### 3.4. Flow Cytometry

Flow cytometry was performed by Lifeasible (New York, NY, USA) following the protocol of the SYTOX^TM^ Green Dead Cell Stain for flow cytometry from Invitrogen (S348960) (San Diego, CA, USA). In brief, 900 μL of PBS was taken into the flow tube; then, thawed bacterial samples were gently mixed, and 100 μL was drawn into the flow tube. Next, 1 μL of the SYTOX^TM^ green dye was added to each tube, vortexed to mix, and incubated for 10 min at room temperature in the dark before subjection to the Cytoflex flow cytometer (Beckman Coulter Life Sciences, Inc., Indianapolis, IN, USA.)

### 3.5. Bioinformatics and Statistical Analysis

Sequence data were processed using QIIME2 [55]. Read pairs were processed to identify amplicon sequence variants with DADA2 [56]. The 285 samples had an average of 44,719 sequences per sample following denoising, representing 1090 unique ASVs. Taxonomic assignments were generated by comparison to the Greengenes reference database (v. 13_8) [57] and using the Silva database (v. 138) [58], using the naive Bayes classifier implemented in scikit-bio [59]. A phylogenetic tree was inferred from the sequence data using MAFFT [60]. Similarity between samples was assessed by the weighted and unweighted UniFrac distance [61,62].

Microbial community functional prediction using 16S rRNA community profiles was performed using PICRUSt2 (version 2.5.1) [63]. Statistical analysis and visualizations were done using R (v.4.1.3) [64] and the packages tidyverse [65] and ggplot2 [66]. The differential estimated enzyme abundance (EC, MetaCyc) was calculated using MaAsLin2 [67]. Statistical analyses were conducted across donors and within donors to account for inter-individual variability.

### 3.6. Transepithelial Electrical Resistance (TEER) Measurement

Caco2 cells were plated in 24-well hanging inserts (cellQART, 0.4 μm pore, polyethylene terephthalate membrane) at a density of 1 × 10^5^ cells/well and allowed to differentiate for 21 days [68]. During this time, cells were maintained in an incubator at 37 °C with 5% CO_2_ in 100 μL growth media (high-glucose DMEM; Gibco + 10% regular fetal bovine serum, Corning + 1X penicillin–streptomycin, Gibco) in the apical chamber and 600 μL in the basolateral chamber. Medium was changed every 2–3 days. Bacteria-free supernatants from the bioreactor representing the proximal colon were collected on Day 1 and Day 4 of IVM treatment for all donors, for all feed types, and were thawed on ice, diluted 1:4 in growth media, and filter-sterilized through a 0.2 μm filter (18 different supernatants total) and used to treat cells. N = 6 wells of cells were treated per treatment condition. Before treatment, plates of cells were removed from the incubator and allowed to equilibrate to RT and then the transepithelial electrical resistance (TEER) was measured with the EVOM2 epithelial voltohmmeter and an STX2 electrode (World Precision Instruments, Sarasota, FL, USA) [68]. Medium was aspirated and cells were rinsed once with PBS before 100 μL of the diluted supernatant was added to the apical chamber and 600 μL of fresh growth medium was added to the basolateral chamber. TEER was measured every 24 h for the next 72 h. TEER readings for each treatment condition and at each timepoint were averaged across donors. Multiple comparisons of a 2-way ANOVA were performed in GraphPad Prism (v.10.0.2) (GraphPad Software, San Diego, CA, USA) to determine significance.

### 3.7. Short-Chain Fatty Acid (SCFA) Determination

SCFA analysis was performed as previously reported [69]. Briefly, samples were thawed immediately before analysis and extracted with ethyl ether (1:1, *v*/*v*). Then, 1 μL extracts were injected into a GC/MS Shimadzu QP2010 Ultra (Shizmadzu, Columbia, MD, USA) equipped with a Stabilwax-DA column (30 m, 0.25 mm ID, 0.25 μm; Restek Corporation, Bellefonte, PA, USA). Analysis was carried out at following settings: split mode, 1:200 at 260 °C, interface temperature, 280 °C; ion source temperature, 220 °C. A standard curve was obtained from a stock solution consisting of the following analytical-grade chemicals at 5 mg/mL of each: acetic acid, butyric acid, propionic acid, 2-methylbutyric acid, isobutyric acid, isopropionic acid, valeric acid, hexanoic acid, heptanoic acid, 2-methylvaleric acid, 3-methylvaleric acid, 4-methylvaleric acid, 2-methylvutyric acid, and isovaleric acid [69]. The data were acquired with a mass range of *m*/*z* 25–375 in full scan mode.

## 4. Conclusions

IVM is often administrated for the treatment of helminth infections, but the recent elucidation of its mechanistic properties has increased its application potential and could extend its use as an antimicrobial or anticancer therapeutic. In this study, the effect of IVM administration on the microbial communities of healthy donors was evaluated and the capacity for nutritional supplementation with dietary fiber to mitigate any effects was examined. Under these experimental conditions, IVM introduced minor, temporary effects on the gut microbial community, assessed in terms of composition and metabolite production. These effects were secondary to established differences in the lumen vs. mucosa and colon regions, as well as to inter-individual differences. In this experiment, IVM was administered directly to the reactor, representing the proximal colon. In practice, IVM would encounter additional microbiota and environmental conditions, potentially further reducing any effects of IVM on the gut microbiome. Taken together, these results imply that short-duration IVM would not be expected to induce dysbiosis or yield adverse effects if administered to healthy adults.

## Data Availability

Raw sequencing data are available in the NCBI Sequence Read Archive under BioProject PRJNA1034049.

## Supplementary Materials

The following supporting information can be downloaded at: https://www.mdpi.com/article/10.3390/ijms242216125/s1. Supplementary Figures S1–S5 and sample information.

## References

[1] Crump A., Omura S. (2011). Ivermectin, ‘wonder drug’ from Japan: The human use perspective. Proc. Jpn. Acad. Ser. B Phys. Biol. Sci..

[2] Roman Y.M., Burela P.A., Pasupuleti V., Piscoya A., Vidal J.E., Hernandez A.V. (2022). Ivermectin for the Treatment of Coronavirus Disease 2019: A Systematic Review and Meta-analysis of Randomized Controlled Trials. Clin. Infect. Dis..

[3] Martin R.J., Robertson A.P., Choudhary S. (2021). Ivermectin: An Anthelmintic, an Insecticide, and Much More. Trends Parasitol..

[4] Sharpton T.J., Combrink L., Arnold H.K., Gaulke C.A., Kent M. (2020). Harnessing the gut microbiome in the fight against anthelminthic drug resistance. Curr. Opin. Microbiol..

[5] Foy B.D., Alout H., Seaman J.A., Rao S., Magalhaes T., Wade M., Parikh S., Soma D.D., Sagna A.B., Fournet F. (2019). Efficacy and risk of harms of repeat ivermectin mass drug administrations for control of malaria (RIMDAMAL): A cluster-randomised trial. Lancet.

[6] Brown K.R., Ricci F.M., Ottesen E.A. (2000). Ivermectin: Effectiveness in lymphatic filariasis. Parasitology.

[7] De Souza D.K., Otchere J., Ahorlu C.S., Adu-Amankwah S., Larbi I.A., Dumashie E., McCarthy F.A., King S.A., Otoo S., Osabutey D. (2018). Low Microfilaremia Levels in Three Districts in Coastal Ghana with at Least 16 Years of Mass Drug Administration and Persistent Transmission of Lymphatic Filariasis. Trop. Med. Infect. Dis..

[8] Byakika-Kibwika P., Kamya M.R., Nankabirwa J. (2022). Ivermectin for mass drug administration against malaria. Lancet Infect. Dis..

[9] Llinas-Caballero K., Caraballo L. (2022). Helminths and Bacterial Microbiota: The Interactions of Two of Humans’ “Old Friends”. Int. J. Mol. Sci..

[10] Rashid M., Rashid M.I., Akbar H., Ahmad L., Hassan M.A., Ashraf K., Saeed K., Gharbi M. (2019). A systematic review on modelling approaches for economic losses studies caused by parasites and their associated diseases in cattle. Parasitology.

[11] Ola-Fadunsin S.D., Ganiyu I.A., Rabiu M., Hussain K., Sanda I.M., Baba A.Y., Furo N.A., Balogun R.B. (2020). Helminth infections of great concern among cattle in Nigeria: Insight to its prevalence, species diversity, patterns of infections and risk factors. Vet. World.

[12] Chen I.S., Kubo Y. (2018). Ivermectin and its target molecules: Shared and unique modulation mechanisms of ion channels and receptors by ivermectin. J. Physiol..

[13] Loghry H.J., Yuan W., Zamanian M., Wheeler N.J., Day T.A., Kimber M.J. (2020). Ivermectin inhibits extracellular vesicle secretion from parasitic nematodes. J. Extracell. Vesicles.

[14] Drurey C., Maizels R.M. (2021). Helminth extracellular vesicles: Interactions with the host immune system. Mol. Immunol..

[15] Zhang X., Song Y., Ci X., An N., Ju Y., Li H., Wang X., Han C., Cui J., Deng X. (2008). Ivermectin inhibits LPS-induced production of inflammatory cytokines and improves LPS-induced survival in mice. Inflamm. Res..

[16] Wagstaff K.M., Sivakumaran H., Heaton S.M., Harrich D., Jans D.A. (2012). Ivermectin is a specific inhibitor of importin alpha/beta-mediated nuclear import able to inhibit replication of HIV-1 and dengue virus. Biochem. J..

[17] Zaidi A.K., Dehgani-Mobaraki P. (2022). The mechanisms of action of ivermectin against SARS-CoV-2-an extensive review. J. Antibiot..

[18] Nabi-Afjadi M., Mohebi F., Zalpoor H., Aziziyan F., Akbari A., Moradi-Sardareh H., Bahreini E., Moeini A.M., Effatpanah H. (2023). A cellular and molecular biology-based update for ivermectin against COVID-19: Is it effective or non-effective?. Inflammopharmacology.

[19] Tang M., Hu X., Wang Y., Yao X., Zhang W., Yu C., Cheng F., Li J., Fang Q. (2021). Ivermectin, a potential anticancer drug derived from an antiparasitic drug. Pharmacol. Res..

[20] Sharmeen S., Skrtic M., Sukhai M.A., Hurren R., Gronda M., Wang X., Fonseca S.B., Sun H., Wood T.E., Ward R. (2010). The antiparasitic agent ivermectin induces chloride-dependent membrane hyperpolarization and cell death in leukemia cells. Blood.

[21] Juarez M., Schcolnik-Cabrera A., Duenas-Gonzalez A. (2018). The multitargeted drug ivermectin: From an antiparasitic agent to a repositioned cancer drug. Am. J. Cancer Res..

[22] Chen L., Bi S., Wei Q., Zhao Z., Wang C., Xie S. (2020). Ivermectin suppresses tumour growth and metastasis through degradation of PAK1 in oesophageal squamous cell carcinoma. J. Cell. Mol. Med..

[23] Hussain R. (2016). Effect of Ivermectin on Pulmonary Aspergillosis in Racing Pigeons. Diyala J. Pure Sci..

[24] Cardwell L.A., Alinia H., Moradi Tuchayi S., Feldman S.R. (2016). New developments in the treatment of rosacea—Role of once-daily ivermectin cream. Clin. Cosmet. Investig. Dermatol..

[25] Hu X., Xu Y., Liu G., Hu D., Wang Y., Zhang W., Zheng Y. (2020). The impact of anthelmintic treatment on gut bacterial and fungal communities in diagnosed parasite-free sika deer Cervus nippon. Appl. Microbiol. Biotechnol..

[26] Miro-Canturri A., Ayerbe-Algaba R., Smani Y. (2019). Drug Repurposing for the Treatment of Bacterial and Fungal Infections. Front. Microbiol..

[27] Shirazi F.M., Mirzaei R., Nakhaee S., Nejatian A., Ghafari S., Mehrpour O. (2022). Repurposing the drug, ivermectin, in COVID-19: Toxicological points of view. Eur. J. Med. Res..

[28] Schneeberger P.H.H., Gueuning M., Welsche S., Hurlimann E., Dommann J., Haberli C., Frey J.E., Sayasone S., Keiser J. (2022). Different gut microbial communities correlate with efficacy of albendazole-ivermectin against soil-transmitted helminthiases. Nat. Commun..

[29] Schneeberger P.H.H., Coulibaly J.T., Gueuning M., Moser W., Coburn B., Frey J.E., Keiser J. (2018). Off-target effects of tribendimidine, tribendimidine plus ivermectin, tribendimidine plus oxantel-pamoate, and albendazole plus oxantel-pamoate on the human gut microbiota. Int. J. Parasitol. Drugs Drug Resist..

[30] Barra N.G., Anhe F.F., Cavallari J.F., Singh A.M., Chan D.Y., Schertzer J.D. (2021). Micronutrients impact the gut microbiota and blood glucose. J. Endocrinol..

[31] Dicks L.M.T., Deane S.M., Grobbelaar M.J. (2022). Could the COVID-19-Driven Increased Use of Ivermectin Lead to Incidents of Imbalanced Gut Microbiota and Dysbiosis?. Probiotics Antimicrob. Proteins.

[32] Van de Wiele T., Van den Abbeele P., Ossieur W., Possemiers S., Marzorati M., Verhoeckx K., Cotter P., Lopez-Exposito I., Kleiveland C., Lea T., Mackie A., Requena T., Swiatecka D., Wichers H. (2015). The Simulator of the Human Intestinal Microbial Ecosystem (SHIME((R))). The Impact of Food Bioactives on Health: In Vitro and Ex Vivo Models.

[33] Liu L., Firrman J., Tanes C., Bittinger K., Thomas-Gahring A., Wu G.D., Van den Abbeele P., Tomasula P.M. (2018). Establishing a mucosal gut microbial community in vitro using an artificial simulator. PLoS ONE.

[34] Piras C., Gugliandolo E., Castagna F., Palma E., Britti D. (2022). Ivermectin (IVM) Possible Side Activities and Implications in Antimicrobial Resistance and Animal Welfare: The Authors’ Perspective. Vet. Sci..

[35] Ashraf S., Chaudhry U., Raza A., Ghosh D., Zhao X. (2018). In vitro activity of ivermectin against Staphylococcus aureus clinical isolates. Antimicrob. Resist. Infect. Control.

[36] Penumutchu S., Korry B.J., Hewlett K., Belenky P. (2023). Fiber supplementation protects from antibiotic-induced gut microbiome dysbiosis by modulating gut redox potential. Nat. Commun..

[37] Muller M., Hermes G.D.A., Canfora E.E., Smidt H., Masclee A.A.M., Zoetendal E.G., Blaak E.E. (2020). Distal colonic transit is linked to gut microbiota diversity and microbial fermentation in humans with slow colonic transit. Am. J. Physiol. Gastrointest. Liver Physiol..

[38] Martinez-Guryn K., Leone V., Chang E.B. (2019). Regional Diversity of the Gastrointestinal Microbiome. Cell Host Microbe.

[39] Louis P., Flint H.J. (2017). Formation of propionate and butyrate by the human colonic microbiota. Environ. Microbiol..

[40] Pedro F.-J., Daniel M.C., Van Douwe S., Jose M.-M. (2022). Cross-feeding interactions between human gut commensals belonging to the *Bacteroides* and *Bifidobacterium* genera when grown on dietary glycans. Microbiome Res. Rep..

[41] Mazhar M., Zhu Y., Qin L. (2023). The Interplay of Dietary Fibers and Intestinal Microbiota Affects Type 2 Diabetes by Generating Short-Chain Fatty Acids. Foods.

[42] Usta-Gorgun B., Yilmaz-Ersan L. (2020). Short-chain fatty acid production by the Bifidobacterium species in the presence of salep. Electron. J. Biotechnol..

[43] Fusco W., Lorenzo M.B., Cintoni M., Porcari S., Rinninella E., Kaitsas F., Lener E., Mele M.C., Gasbarrini A., Collado M.C. (2023). Short-Chain Fatty-Acid-Producing Bacteria: Key Components of the Human Gut Microbiota. Nutrients.

[44] Christopherson M.R., Dawson J.A., Stevenson D.M., Cunningham A.C., Bramhacharya S., Weimer P.J., Kendziorski C., Suen G. (2014). Unique aspects of fiber degradation by the ruminal ethanologen Ruminococcus albus 7 revealed by physiological and transcriptomic analysis. BMC Genom..

[45] Leschine S.B. (1995). Cellulose degradation in anaerobic environments. Annu. Rev. Microbiol..

[46] Dilnessa T., Getaneh A., Hailu W., Moges F., Gelaw B. (2022). Prevalence and antimicrobial resistance pattern of Clostridium difficile among hospitalized diarrheal patients: A systematic review and meta-analysis. PLoS ONE.

[47] Tomas-Pejo E., Gonzalez-Fernandez C., Greses S., Kennes C., Otero-Logilde N., Veiga M.C., Bolzonella D., Muller B., Passoth V. (2023). Production of short-chain fatty acids (SCFAs) as chemicals or substrates for microbes to obtain biochemicals. Biotechnol. Biofuels Bioprod..

[48] Van den Abbeele P., Roos S., Eeckhaut V., MacKenzie D.A., Derde M., Verstraete W., Marzorati M., Possemiers S., Vanhoecke B., Van Immerseel F. (2012). Incorporating a mucosal environment in a dynamic gut model results in a more representative colonization by lactobacilli. Microb. Biotechnol..

[49] Firrman J., Liu L., Van den Abbeele P., Tanes C., Bittinger K., Tomasula P. (2019). Applying Advanced In Vitro Culturing Technology to Study the Human Gut Microbiota. J. Vis. Exp..

[50] Mahalak K.K., Firrman J., Lee J.J., Bittinger K., Nunez A., Mattei L.M., Zhang H., Fett B., Bobokalonov J., Arango-Argoty G. (2020). Triclosan has a robust, yet reversible impact on human gut microbial composition in vitro. PLoS ONE.

[51] Mayo Clinic Ivermectin (Oral Route). https://www.mayoclinic.org/drugs-supplements/ivermectin-oral-route/proper-use/drg-20064397.

[52] Laffont C., Toutain P.L., Alvinerie M., Bousquet-Mélou A. (2002). Intestinal secretion is a major route for parent ivermectin elimination in the rat. Drug Metab. Dispos..

[53] Cesar T., Salgaco M.K., Mesa V., Sartoratto A., Sivieri K. (2023). Exploring the Association between Citrus Nutraceutical Eriocitrin and Metformin for Improving Pre-Diabetes in a Dynamic Microbiome Model. Pharmaceuticals.

[54] Mahalak K.K., Firrman J., Bobokalonov J., Narrowe A.B., Bittinger K., Daniel S., Tanes C., Mattei L.M., Zeng W.B., Soares J.W. (2022). Persistence of the Probiotic Lacticaseibacillus rhamnosus Strain GG (LGG) in an In Vitro Model of the Gut Microbiome. Int. J. Mol. Sci..

[55] Bolyen E., Rideout J.R., Dillon M.R., Bokulich N.A., Abnet C.C., Al-Ghalith G.A., Alexander H., Alm E.J., Arumugam M., Asnicar F. (2019). Reproducible, interactive, scalable and extensible microbiome data science using QIIME 2. Nat. Biotechnol..

[56] Callahan B.J., McMurdie P.J., Rosen M.J., Han A.W., Johnson A.J., Holmes S.P. (2016). DADA2: High-resolution sample inference from Illumina amplicon data. Nat. Methods.

[57] McDonald D., Price M.N., Goodrich J., Nawrocki E.P., DeSantis T.Z., Probst A., Andersen G.L., Knight R., Hugenholtz P. (2012). An improved Greengenes taxonomy with explicit ranks for ecological and evolutionary analyses of bacteria and archaea. ISME J..

[58] Quast C., Pruesse E., Yilmaz P., Gerken J., Schweer T., Yarza P., Peplies J., Glockner F.O. (2013). The SILVA ribosomal RNA gene database project: Improved data processing and web-based tools. Nucleic Acids Res..

[59] Bokulich N.A., Kaehler B.D., Rideout J.R., Dillon M., Bolyen E., Knight R., Huttley G.A., Gregory Caporaso J. (2018). Optimizing taxonomic classification of marker-gene amplicon sequences with QIIME 2’s q2-feature-classifier plugin. Microbiome.

[60] Katoh K., Standley D.M. (2013). MAFFT multiple sequence alignment software version 7: Improvements in performance and usability. Mol. Biol. Evol..

[61] Lozupone C., Knight R. (2005). UniFrac: A new phylogenetic method for comparing microbial communities. Appl. Environ. Microbiol..

[62] Lozupone C.A., Hamady M., Kelley S.T., Knight R. (2007). Quantitative and qualitative beta diversity measures lead to different insights into factors that structure microbial communities. Appl. Environ. Microbiol..

[63] Douglas G.M., Maffei V.J., Zaneveld J.R., Yurgel S.N., Brown J.R., Taylor C.M., Huttenhower C., Langille M.G.I. (2020). PICRUSt2 for prediction of metagenome functions. Nat. Biotechnol..

[64] R Core Team (2022). R: A Language and Environment for Statistical Computing.

[65] Wickham H., Averick M., Bryan J., Chang W., D’Agostino McGowan L., François R., Garrett Grolemund G., Hayes A., Henry L., Hester J. (2019). Welcome to the Tidyverse. J. Open Source Softw..

[66] Wickham H. (2016). ggplot2: Elegant Graphics for Data Analysis.

[67] Mallick H., Rahnavard A., McIver L.J., Ma S., Zhang Y., Nguyen L.H., Tickle T.L., Weingart G., Ren B., Schwager E.H. (2021). Multivariable association discovery in population-scale meta-omics studies. PLOS Comput. Biol..

[68] Srinivasan B., Kolli A.R., Esch M.B., Abaci H.E., Shuler M.L., Hickman J.J. (2015). TEER measurement techniques for in vitro barrier model systems. J. Lab. Autom..

[69] Mahalak K.K., Bobokalonov J., Firrman J., Williams R., Evans B., Fanelli B., Soares J.W., Kobori M., Liu L. (2022). Analysis of the Ability of Capsaicin to Modulate the Human Gut Microbiota In Vitro. Nutrients.

