# Supplementary material for: Impact of Ivermectin on the Gut Microbial Ecosystem
Source: Int J Mol Sci. 2023 Nov 9;24(22):16125. doi: 10.3390/ijms242216125 (PMC10671733; doi:10.3390/ijms242216125)
Supplement: Supplementary file 1 [file ijms-24-16125-s001.zip › Supplemental_Figures_for_ijms-2678133.pdf]

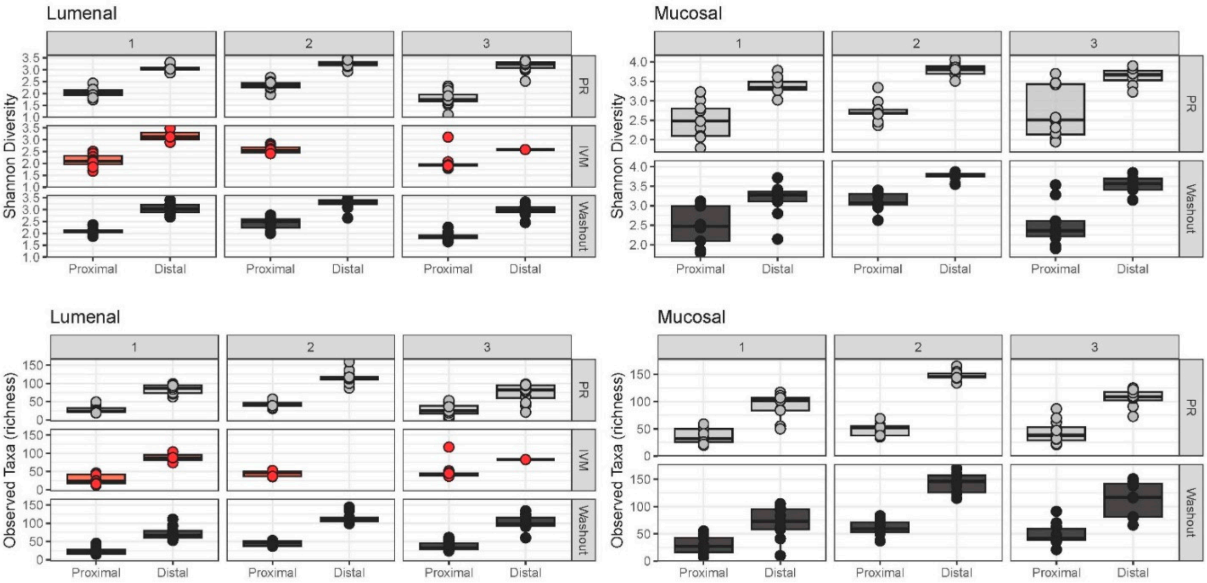

Figure S1. Alpha diversity in terms of observed taxa (richness) and evenness (Shannon's index) for the luminal and mucosal communities.

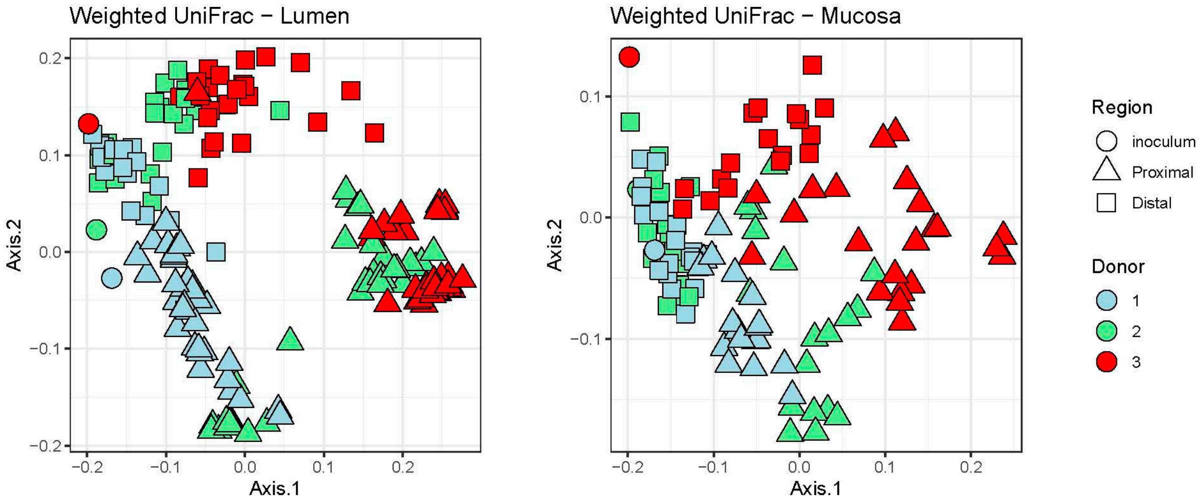

Figure S2. Weighted UniFrac distances portrayed as Principal Coordinates Analysis (PCoA) for the luminal and mucosal communities of each donor for the inoculum, proximal and distal colon regions.

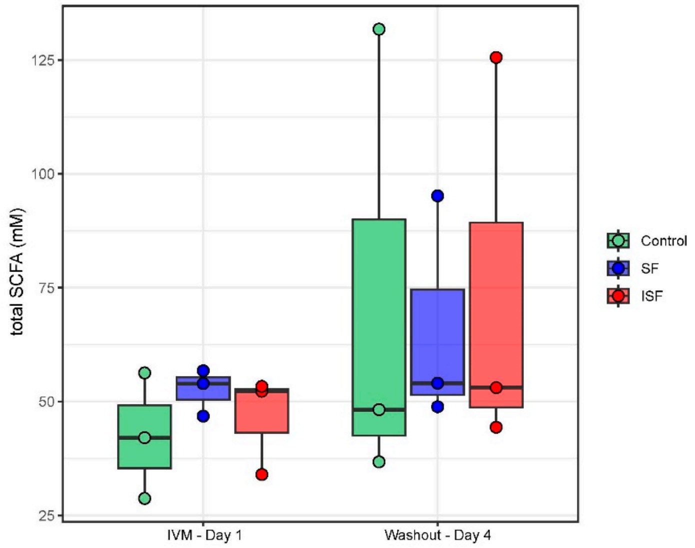

Figure S3. Concentrations of SCFAs from samples used in TEER experiment (Figure 6.).

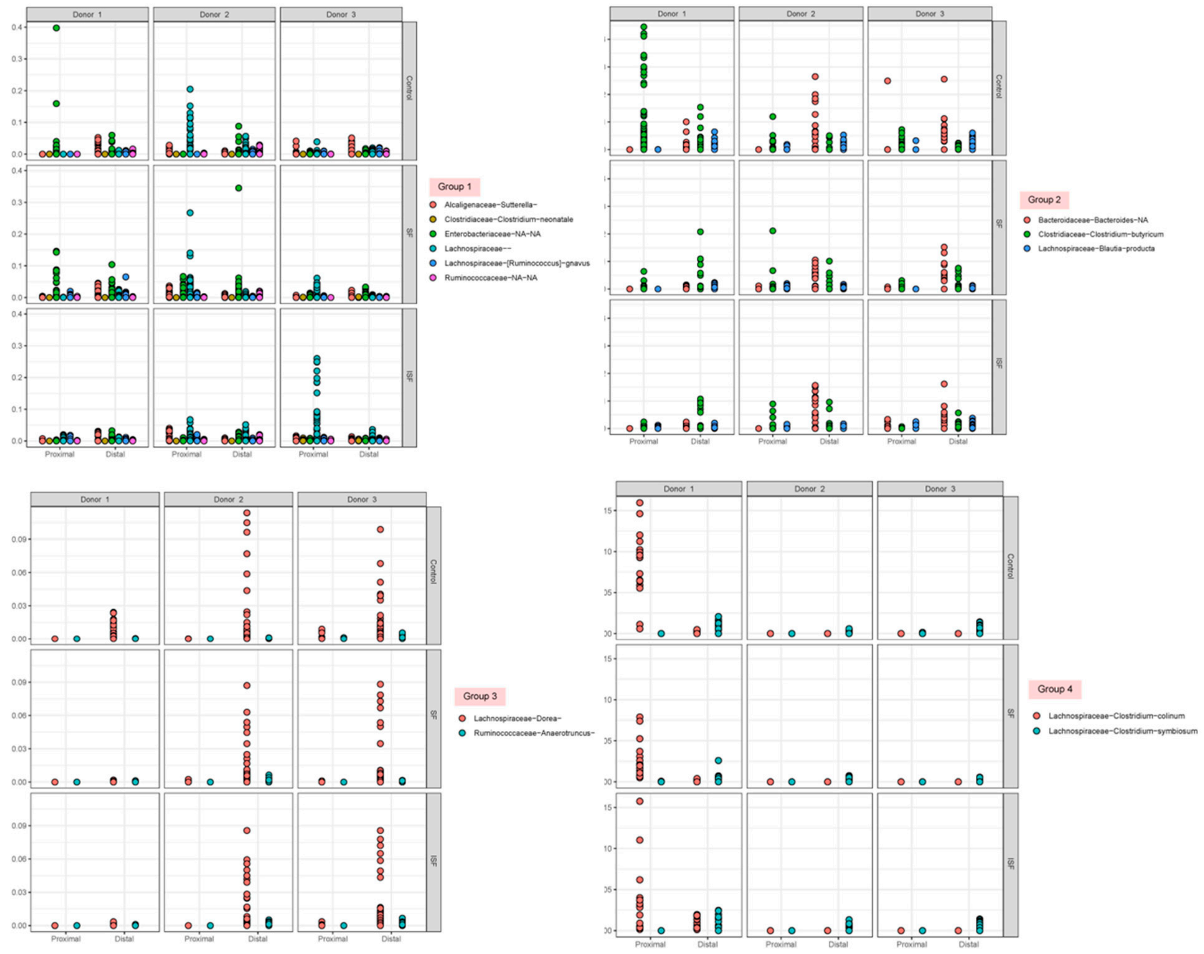

Figure S4. Relative abundances of taxa predicted to contribute cellulase EC 3.2.1.4. Group identity refers to Figure 8.

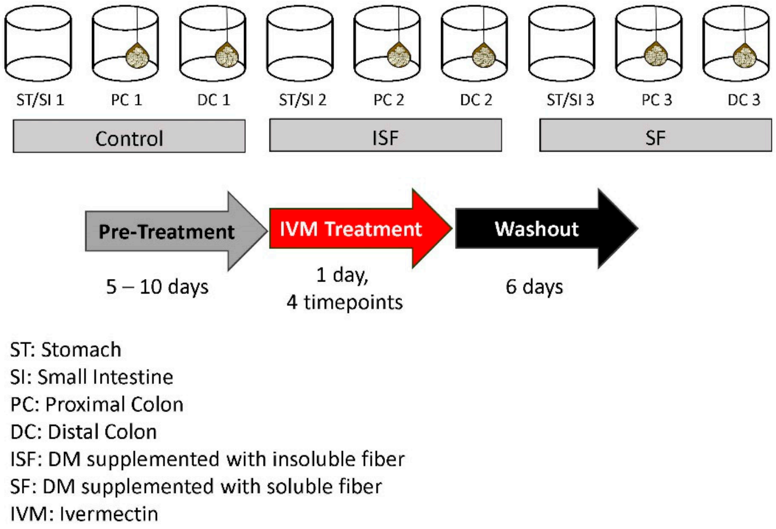

Figure S5. Schematic showing experimental design.
